# Depression in Autism Spectrum Disorder: Neurobiological Convergence and Emerging Therapeutic Strategies

**DOI:** 10.3390/biology15100745

**Published:** 2026-05-08

**Authors:** Seham M. Al Raish, Mustafa M. Shokr, Reem M. Eladawy, Yasmena O. Azar

**Affiliations:** 1 Department of Biology, College of Science, United Arab Emirates University, Al Ain 15551, United Arab Emirates; 2 Department of Pharmacology and Toxicology, Faculty of Pharmacy, Sinai University—Arish Branch, Arish 45511, Egypt

**Keywords:** autism spectrum disorder, major depressive disorder, neuroinflammatory diseases, brain–gut axis, probiotics, phytotherapy, precision medicine

## Abstract

The high comorbidity between autism spectrum disorder (ASD) and major depressive disorder (MDD) represents a complex and heterogeneous clinical challenge. Although elevated rates of depression in autistic individuals are well documented, the neurobiological and psychosocial mechanisms underlying this overlap remain debated, particularly in adulthood. This review synthesizes convergent evidence from genetics, monoaminergic and glutamatergic neurotransmission, neuroinflammatory signaling, hypothalamic–pituitary–adrenal axis dysregulation, large-scale brain network alterations, and gut–brain axis modulation to clarify the biological and psychosocial pathways contributing to ASD–depression comorbidity. In addition to shared neurobiological vulnerability, cumulative environmental stressors such as chronic social masking, stigma, and structural barriers may amplify depressive risk across the lifespan. We propose a hypothesis-generating framework that integrates these findings into a mechanistic stratification model, linking dominant biological profiles to targeted therapeutic hypotheses. Within this preliminary model, plant-derived compounds and microbiota-targeted interventions are hypothesized to serve as promising adjunctive strategies, particularly in neuroinflammatory and stress-related subtypes, complementing established pharmacological and neuromodulatory treatments. By moving beyond descriptive overlap toward biologically informed stratification, this review aims to support precision-guided and neurodiversity-affirming approaches for the assessment and treatment of depression in autistic individuals, especially adults who bear a disproportionate burden of mood disorders. This narrative review is based on a structured literature search conducted in PubMed, Scopus, and Web of Science using combinations of keywords such as “autism spectrum disorder,” “major depressive disorder,” “neuroinflammation,” and “gut–brain axis.

## Simple Summary

Depression occurs at disproportionately high rates in individuals with autism spectrum disorder (ASD), particularly in adulthood. This comorbidity arises from a bidirectional interplay where shared neurobiological vulnerabilities including genetic overlap, monoaminergic dysregulation, and neuroinflammation are exacerbated by cumulative psychosocial stressors. These shared vulnerabilities include overlapping genetic factors, disruptions in serotonin and dopamine signaling, and neuroinflammation. Moreover, alterations in brain networks involved in emotion regulation may increase susceptibility to depressive symptoms. At the same time, chronic social stress, masking behaviors, and structural barriers can further amplify risk across the lifespan. Depression in autistic individuals often presents atypically, making accurate diagnosis challenging and increasing the likelihood of under-recognition or misinterpretation. Conventional treatments such as selective serotonin reuptake inhibitors and other antidepressants may show variable effectiveness in this population. Emerging approaches, including glutamatergic modulators, transcranial magnetic stimulation, plant-derived compounds, and microbiota-targeted interventions, may offer promising complementary strategies, as preliminary evidence suggests. This review proposes an integrative framework to better understand the biological diversity underlying ASD–depression comorbidity and to guide more personalized and neurodiversity-informed treatment approaches.

## 1. Introduction

### 1.1. Epidemiology

According to current studies, there is a disproportionately high occurrence of depressive disorders among autistic people compared to the general population, making the junction of depression and autism spectrum disorder (ASD) a major and compounding problem in mental health care [1]. According to recent research, up to 40.2% of adults with autism had a lifetime diagnosis of depression, which is around four times greater than the rate for their neurotypical peers [2,3]. This comorbidity is not a coincidental discovery; rather, it is the result of a confluence of social and autism related elements [2]. ASD’s sensory sensitivity, executive dysfunction, and cognitive rigidity can all lead to increased stress and anxiety reactions, which may eventually result in depressive episodes [4].

### 1.2. Diagnostic Challenges

Alexithymia, the inability to identify and categorize emotions, can mask the feeling of depression in people with autism, making it more difficult for them to recognize and express sadness and making it more difficult for medical professionals to diagnose them accurately [5]. Clinical detection is often hampered by diagnostic overshadowing, where depressive symptoms are misattributed exclusively to autism [5]. This is exacerbated by atypical presentations, such as increased irritability, intensified ritualistic behaviors, or the loss of acquired skills, which deviate from neurotypical diagnostic markers [6]. There are significant diagnostic challenges because of this unusual presentation, communication difficulties, and the paucity of standardized instruments validated specifically for autistic populations [6]. Comorbid depression has devastating effects on autistic patients, including worsening of pre-existing impairments related to ASD, increased rates of self-harm and suicidal thoughts, and a reduced quality of life [1]. These effects highlight the urgent need for individualized and interprofessional care approaches [1]. Effective therapy typically includes an integrated understanding of how neurodevelopmental differences intersect with mental health issues, with adaptations of treatments such as cognitive behavioral therapy (CBT) to fit the communication and information processing patterns of autistic people [7]. Greater sensitivity, additional training for clinicians, and the creation of neurodiversity-affirming diagnostic instruments and therapies attuned to the particular needs of autistic people are all necessary to navigate this cross-sectional landscape, thereby promoting better mental health and general well-being.

### 1.3. Contributing Factors

The multifactorial cause of the increased risk results from the interaction of environmental factors and the innate traits of autism [8]. Severe social difficulties, such as social isolation, bullying, and an overpowering sensation of loneliness, are common in people with autism and are known risk factors for depression [8]. The persistent attempt to hide autistic characteristics to blend in with a neurotypical setting can result in persistent stress, burnout, and a loss of identity, all of which have a detrimental impact on mental health outcomes [8]. Growing evidence reveals significant genetic overlap between ASD and MDD, suggesting shared inherent vulnerabilities [9,10]. A critical analysis of this overlap, however, reveals that it is highly pleiotropic; depending on environmental triggers or epigenetic alterations, the same genetic variants may result in different clinical symptoms. Although shared loci have been found by large-scale GWAS, only a small portion of the heritable risk is now explained by these discoveries. This indicates that comorbidity cannot be predicted just by genetic architecture, underscoring the critical role that gene-environment interactions play in the development of depression in the autistic community [11,12].

### 1.4. Clinical Implications and Review Objectives

Unlike previous reviews that have focused independently on autistic traits and psychosocial predictors of depressive symptoms [13], or more broadly on neurobiological relationships between neurodevelopmental and mood disorders [9], this review provides: (1) a unified neurobiology-psychosocial-microbiota framework; (2) an adult ASD stratification model; and (3) a precision-oriented intervention roadmap that differentiates the present review from prior, more isolated analyses.

## 2. Methodology

This narrative review was conducted to comprehensively synthesize current evidence on the neurobiological and psychosocial mechanisms underlying the comorbidity between autism spectrum disorder (ASD) and major depressive disorder (MDD). To enhance transparency and reproducibility, a structured literature search strategy was employed.

Electronic databases including PubMed, Scopus, and Web of Science were systematically searched for relevant studies published from January 2000 to 31 December 2025. The search strategy combined controlled vocabulary (e.g., MeSH terms) and free-text keywords, including: “autism spectrum disorder,” “depression,” “major depressive disorder,” “neuroinflammation,” “HPA axis,” “gut–brain axis,” “microbiota,” “psychobiotics,” “antidepressants,” “plant-derived compounds,” and “precision psychiatry.” Boolean operators (AND/OR) were used to refine the search.

Studies were included based on the following criteria:

(i) Relevance to ASD–depression comorbidity;
(ii) Focus on neurobiological, psychosocial, or therapeutic mechanisms;
(iii) Publication in peer-reviewed journals;
(iv) Availability in English.

Priority was given to systematic reviews, meta-analyses, randomized controlled trials, and high-quality observational studies, while key mechanistic and preclinical studies were included where necessary to support biological interpretation.

Articles were screened based on titles and abstracts, followed by full-text evaluation for eligibility. Reference lists of selected articles were also manually screened to identify additional relevant studies.

Given the narrative nature of this review, a formal risk-of-bias assessment was not performed; however, findings were critically interpreted with consideration of study design, sample size, and methodological limitations. Importantly, human clinical evidence is distinguished from preclinical findings throughout the manuscript to enhance translational relevance and avoid overinterpretation.

## 3. Current Debate on the Nature of ASD–Depression Overlap

Despite the growing recognition that depression is one of the most common psychiatric comorbidities in individuals with ASD, the precise nature of the relationship between these conditions remains a subject of considerable debate within the autism research community [11]. Several competing theoretical frameworks have been proposed to explain the high prevalence of depressive symptoms among autistic individuals, particularly in adulthood [11].

### 3.1. Shared Biological Vulnerability Model

One influential perspective suggests that ASD and depression share underlying biological vulnerabilities, including overlapping genetic susceptibility, dysregulation of monoaminergic neurotransmission, neuroinflammatory signaling, and alterations in neural circuits involved in reward processing and emotional regulation [1].

Evidence from genetic and neuroimaging studies supports partially shared biological pathways between ASD and depression, including convergent molecular signaling and circuit-level alterations [1,12]. However, the magnitude of these associations is generally modest. Effect sizes identified in genome-wide association studies are typically small (odds ratios ~1.05–1.2 for common variants) with larger effects observed primarily in rare variants, indicating that shared vulnerability is probabilistic rather than deterministic. Importantly, these findings should be interpreted with caution due to several limitations. Many studies rely on heterogeneous cohorts, cross-sectional designs, and varying diagnostic criteria, which may introduce bias and limit comparability. Additionally, the polygenic architecture of both ASD and MDD, along with gene–environment interactions, complicates causal inference [14,15,16]. Neuroimaging studies are further constrained by small sample sizes and variability in analytic methods, which may reduce reproducibility [1,12].

**Figure 1:**
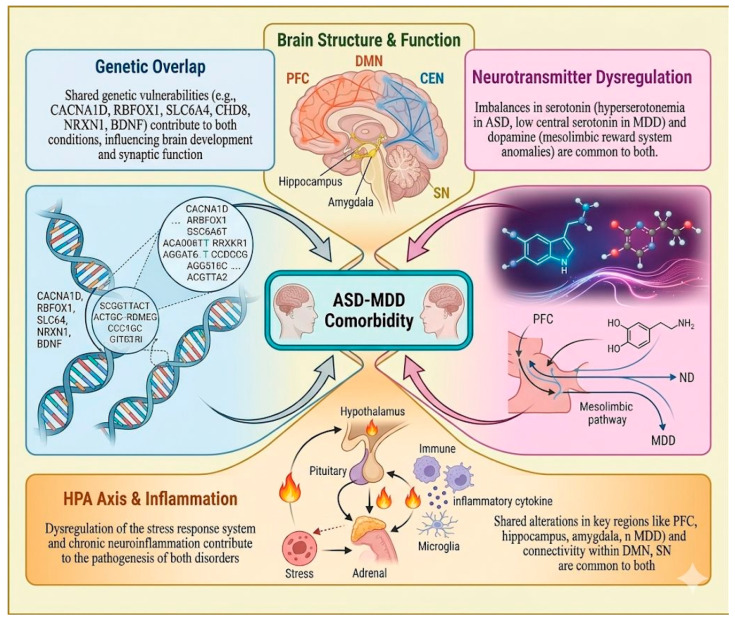
Intersecting Neurobiological Pathways in Autism Spectrum Disorder (ASD) and Major Depressive Disorder (MDD) Comorbidity. This figure illustrates the shared neurobiological vulnerabilities contributing to the high comorbidity between ASD and MDD. It highlights four key intersecting pathways: “Genetic Overlap” (e.g., CACNA1D, RBFOX1, SLC6A4, CHD8, NRXN1, BDNF), “Neurotransmitter Dysregulation” (imbalances in serotonin and dopamine), “HPA Axis & Inflammation” (dysregulation of the stress response system and chronic neuroinflammation), and “Brain Structure & Function” (shared alterations in regions like the Prefrontal Cortex (PFC), hippocampus, amygdala, and functional connectivity within Default Mode Network (DMN), Central Executive Network (CEN), and Salience Network (SN)). These convergent mechanisms underscore the complex interplay driving the co-occurrence of these disorders.

The genetic and molecular components of this shared vulnerability are summarized in Table 1, which outlines convergent risk variants and pathways implicated in both ASD and MDD. Furthermore, the broader neurobiological intersections between these conditions, including neurotransmitter dysregulation, HPA axis alterations, inflammatory mechanisms, and network-level changes, are illustrated in Figure 1, highlighting the convergent pathways that may underlie this comorbidity.

Within this framework, depression in ASD is conceptualized as a true psychiatric comorbidity arising from shared neurobiological risk architecture. The convergence of genetic factors, neurotransmitter dysregulation, and immune-related mechanisms may therefore increase vulnerability to depressive disorders across the lifespan.

### 3.2. Chronic Stress and Environmental Burden Model

A second explanatory framework emphasizes the role of chronic environmental stressors associated with living in a predominantly neurotypical society [13]. According to this perspective, depression in ASD arises primarily as a consequence of cumulative psychosocial adversity rather than intrinsic biological overlap alone [13]. Experiences such as persistent social exclusion, bullying, stigma, employment difficulties, and the cognitive burden associated with masking or camouflaging autistic traits may collectively contribute to long-term psychological distress and increased vulnerability to depressive disorders [13]. Mechanistically, chronic exposure to such stressors activates the HPA axis, leading to sustained cortisol elevation and, over time, glucocorticoid resistance; concurrently, stress-induced sympathetic activation and microglial priming promote pro-inflammatory cytokine release (IL-6, TNF-α), both of which are well-established pathways to major depressive disorder [17,18,19].

In this model, depression is understood as a stress-mediated outcome of sustained social and environmental strain. Particularly in adulthood, prolonged exposure to these stressors may interact with the neurobiological vulnerabilities summarized in Figure 1, amplifying risk for mood dysregulation. Crucially, these two frameworks are not mutually exclusive; rather, long-term psychosocial stress can intensify pre-existing neurobiological vulnerabilities (such as neuroinflammation and dysregulation of the HPA axis), resulting in a reciprocal and self-reinforcing relationship between biological susceptibility and environmental burden. Therefore, it is better to think of the shared vulnerability and chronic stress models as complementary rather than opposing explanations.

### 3.3. Diagnostic and Methodological Model

A third perspective highlights methodological and diagnostic challenges in identifying depression within autistic populations [9]. Many commonly used depression assessment tools were developed for neurotypical individuals and may not adequately capture the atypical emotional expression, alexithymia, or communication differences frequently observed in ASD [9].

As a result, depressive symptoms may be either underrecognized or misinterpreted, complicating accurate prevalence estimates and clinical evaluation [9]. This raises the possibility that part of the observed overlap reflects diagnostic artifact or measurement limitations rather than solely true biological comorbidity.

Whether the higher rates of depression in ASD are mostly caused by measurement bias or actually reflect comorbidity is a crucial subject. The information that is now available indicates that both elements play a role, although the relative importance of each varies depending on the situation [20]. In clinical settings, false negative rates (i.e., missed or delayed diagnoses of depression) are likely to rise due to diagnostic overshadowing and alexithymia-related underreporting, potentially underestimating true prevalence [21,22]. However, population-based research that use formal clinical interviews, which help to partially offset these biases, consistently find that autistic adults have three to four times higher rates of depression than neurotypical adults [23]. This consistency across several approaches indicates a significant level of real biological and environmental comorbidity. The observed epidemiological trends are not entirely explained by measurement problems, albeit they may account for some of the variance, especially in milder or atypical depressed presentations. To further separate these contributions, further research using multi-informant assessment techniques and diagnostic tools tailored to autism will be crucial.

### 3.4. Integrative Perspective

**Figure 2:**
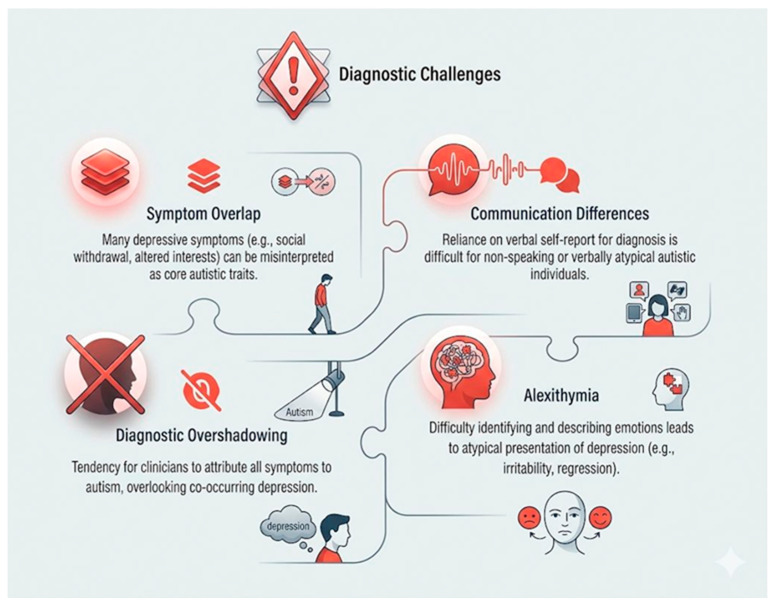
Diagnostic Challenges in Recognizing Depression in Autistic Individuals. This figure highlights the significant obstacles clinicians face when diagnosing major depressive disorder (MDD) in people with autism spectrum disorder (ASD). Key challenges include “Symptom Overlap,” where typical depressive signs are misinterpreted as core autistic traits; “Communication Differences,” which complicate reliance on verbal self-report; “Alexithymia,” leading to difficulty identifying and describing emotions, resulting in atypical depression presentations (e.g., irritability, regression); and “Diagnostic Overshadowing,” a tendency to attribute all symptoms solely to autism, thereby delaying proper diagnosis and intervention. These factors collectively contribute to the under-recognition and undertreatment of depression in the autistic population.

These competing interpretations illustrate the complexity of disentangling biological comorbidity from environmental influences and diagnostic artifacts. At a mechanistic level, HPA axis dysregulation and neuroinflammation should not be viewed as separate parallel processes only; chronic stress can amplify inflammatory signaling, and inflammatory activation can in turn reinforce stress-system dysfunction, together increasing vulnerability to depressive symptoms. Rather than being mutually exclusive, these frameworks may operate simultaneously, with shared neurobiological vulnerability (Table 1; Figure 1 and Figure 2) interacting dynamically with chronic psychosocial stress and assessment challenges. The intersections between frameworks are particularly significant: prolonged environmental stress can worsen neuroinflammatory and HPA axis dysfunction, while biological vulnerability may enhance sensitivity to stress-induced mood dysregulation. Similarly, by hindering emotion detection and help-seeking behavior, diagnostic difficulties (such as alexithymia) may increase the psychological impact of social stressors [24].

Addressing this debate is crucial for advancing the field, as clarifying the mechanisms underlying ASD–depression overlap will inform the development of more accurate diagnostic approaches and more targeted therapeutic strategies for autistic individuals across the lifespan, particularly for adults who experience a disproportionately high burden of mood disorders.

**Table 1:** Summary of shared genetic variants and pathways in ASD and MDD.

Gene	Function	ASD Evidence	Depression Evidence	Mechanistic Overlap	Representative Effect/Estimate	Evidence Confidence	References
CACNA1D (CaV1.2)	Voltage gated calcium channel subunit	De novo mutations linked to ASD; regulates synaptic plasticity and neuronal excitability	GWAS associations with MDD; modulates stress responsive circuits in limbic regions	Dysregulated calcium signaling impairs synaptic pruning and stress adaptation	Common-variant psychiatric effects are generally modest (typical GWAS OR ~1.05–1.2); ASD evidence is stronger for rare/de novo variants.	Moderate	[25]
RBFOX1	RNA splicing regulator in neurons	CNVs associated with ASD; regulates synaptic gene networks	GWAS hits for MDD; RBFOX1 knockdown reduces dendritic complexity	Shared disruption of synaptic maturation and neural connectivity	Association reported across CNV/GWAS studies; no directly comparable pooled effect estimate available.	Moderate	[26]
SLC6A4 Serotonin transporter (SERT)	Serotonin transporter	25–30% of ASD cases show hyperserotonemia; SERT polymorphisms alter social behavior	Short allele (5-HTTLPR) increases stress sensitivity and MDD risk	Serotonin dysregulation affects mood, social cognition, and stress response	Hyperserotonemia reported in ~25–30% of ASD cases; 5-HTTLPR effects are modest and context-dependent.	Moderate	[27]
CHD8	Chromatin remodeler	High penetrance ASD mutations; regulates Wnt/β catenin signaling	CHD8+/− mice show depressive-like behaviors	Epigenetic dysregulation of neurodevelopmental pathways	Rare, high-penetrance ASD mutations; depression link is supported mainly by preclinical data.	High for ASD; low-to-moderate for depression	[28]
NRXN1	Presynaptic cell adhesion molecule	CNVs linked to ASD and schizophrenia	NRXN1 deletions increase MDD risk 2.5 fold	Impaired synaptic vesicle release and neural circuit formation	NRXN1 deletions associated with ~2.5-fold increased MDD risk.	Moderate	[29]
BDNF	Neurotrophins regulating synaptic plasticity	Reduced BDNF in ASD cortex and serum	Val66Met polymorphism predicts poor SSRI response	Shared deficits in neurogenesis and synaptic maintenance	Val66Met associations are variable across studies and are more consistent for treatment response than for primary disease risk.	Moderate	[30]

### 3.5. Neurotransmitter Systems: Serotonin and Dopamine Dysregulation

Serotonin and dopamine are two important neurotransmitter systems that exhibit severe dysregulation in the complex neuroanatomy of the neurobiological mechanisms underlying MDD and ASD [9] (Figure 1). These systems underlie shared symptomatology and account for the high comorbidity between the two disorders. The monoamine neurotransmitter serotonin (5-hydroxytryptamine, 5-HT), which is essential for mood, food, sleep, and social behavior, has a complicated and perhaps conflicting role in autism [31]. Approximately 25% of autistic individuals exhibit hyperserotonemia, meaning elevated serotonin in whole blood, one of the earliest peripheral biomarkers reported in ASD [27]. Importantly, peripheral serotonin refers to serotonin measured outside the central nervous system, whereas central serotonin refers to serotonin signaling within the brain; these are not interchangeable because blood serotonin does not cross the blood–brain barrier [27,32]. Accordingly, elevated peripheral serotonin in ASD does not necessarily imply increased central serotonergic activity. In contrast, imaging and tryptophan-depletion studies suggest that some autistic individuals may have reduced central serotonin availability or altered receptor binding, which may contribute to irritability and repetitive behaviors [33]. In depression, the more widely discussed abnormality involves altered central serotonergic signaling in brain circuits related to mood, sleep, and reward [33,34]. This distinction helps explain why peripheral and central serotonin findings may appear divergent while still pointing to serotonergic dysregulation as a shared, but biologically complex, feature of ASD–depression overlap [35].

Dopamine is another significant monoamine neurotransmitter that plays a major role in executive processes, motor control, motivation, reward, and pleasure processing. Dopamine dysregulation is becoming more well recognized as a shared neurobiological mechanism underpinning depression and ASD [36]. Autism is associated with anomalies in the mesolimbic dopamine reward system, which may result in the characteristic restricted and repetitive behaviors, anhedonia (the inability to experience pleasure), and decreased motivation [37]. For example, an overreliance on safe, self-inducing behaviors or intense, specialized hobbies that offer a safe but constrained source of dopamine release, and less attention to novel or intricate social rewards, can result from an overactive reward system [37]. Common symptoms of severe depressive disorder, such as anhedonia, lack of desire, and motor performance impairment, are intimately linked to decreased dopamine activity, especially in reward circuits [37]. Although both conditions are characterized by dopamine dysregulation, the syndromes can differ; in autism, it may be the cause of rigid special interests or repetitive behaviors, which are more characteristic of ASD; in depression, it is mainly responsible for the widespread loss of motivation and enjoyment [38].

Research shows disruptions in complex neuronal signaling across a wide range of mesolimbic regions, neurotransmitters, and receptor subtypes, but the exact mechanisms by which aberrant dopamine signaling contributes to the multifactorial etiologies of ASD and its comorbidity with depression remain under investigation [39]. Optimizing target-specific pharmacological and behavioral interventions will require a thorough understanding of these pervasive and distinctive patterns of serotonin and dopamine dysregulation [40]. Treatments that alter these neurotransmitter systems, like SSRIs or other psychotropics, for instance, may be beneficial for treating comorbid symptoms, but they must be used carefully with autistic populations, taking into account their unique neurobiological profiles and potential for unusual reactions [41]. Last but not least, a clear comprehension of these neurotransmitter abnormalities provides very relevant insights into the complex pathophysiology of ASD and depression, which may lead to more targeted and efficient treatment strategies that target the neurobiological weaknesses.

### 3.6. Brain Structure and Function: Shared Alterations in Key Brain Regions

The substantial comorbidity of ASD and MDD is being explained by neuroimaging studies that show increasingly intricate topographies of common alterations in brain structure and function [42] (Table 2, Figure 1). Atypicalities in the areas most important to executive function, social cognition, and emotional processing have been structurally linked to both depression and ASD [43]. For example, both ASD and MDD show alterations in hippocampal and amygdala structure and function compared with neurotypical controls, but in opposite directions: early enlargement in ASD versus volume loss in chronic MDD, and differential patterns of emotional reactivity [44]. Both illnesses frequently show anatomical and functional imbalances in the prefrontal cortex (PFC), particularly within subregions implicated in executive processes, decision making, and mood regulation. The anterior cingulate cortex and the orbitofrontal cortex are two regions consistently linked to the pathophysiology of depression [45]. Abnormalities in grey matter volume have been observed in the frontal, temporal, and parietal lobes of people with ASD [46]. Subsequent studies have discovered that patients with autism have fewer synapses throughout their brains than neurotypicals, and that the degree of autistic features is correlated with synaptic density [47,48]. This finding may be crucial in understanding how vulnerable autistic people are to other disorders, such as depression.

Large-scale disturbances in brain networks are a remarkably common functional characteristic [49]. Abnormal functional connectivity between and within networks, including the central executive network (CEN), salience network (SN), and default mode network (DMN), is a common feature of both ASD and depression [49]. Both depression and ASD have a propensity to exhibit aberrantly altered connectivity in the DMN, which is involved in mind wandering and self-referential cognition. This can lead to rumination in depression and social cognition impairment in autism [49]. Furthermore, it is increasingly recognized that the cerebellum, once thought to be motor-specific, also contributes to higher cognition and emotional processing. Both depression and ASD exhibit structural and functional abnormalities in this area [50]. White matter tracts, which are essential for effective information processing and include the cingulum and superior longitudinal fasciculus, have also been found to be disrupted in ASD [51]. Although the unique diagnostic characteristics of each disorder are based on individual manifestations of these brain differences, their convergence offers a crucial neurobiological basis for the high rates of comorbidity and opens up promising directions for transdiagnostic research and integrative treatment approaches that focus on these convergent brain mechanisms [52].

**Table 2:** Neuroimaging findings in ASD and MDD.

Brain Region/Network	ASD Findings	MDD Findings	Convergent Mechanisms	Divergences	References
DLPFC	8% reduced gray matter volume Hypoactivation during executive tasks	12% volume loss in chronic MDD Reduced functional coupling with the amygdala	Impaired top down emotional regulation	ASD: Early overgrowth, then pruning MDD: Stress-induced atrophy	[42,44,45]
Amygdala	6% enlargement in childhood ASD Hyperreactivity to neutral faces	9% volume loss in recurrent MDD Hyperreactivity to negative stimuli	Limbic hyperarousal	ASD: Developmental timing differences MDD: Glucocorticoid mediated damage	[44,53]
Hippocampus	Early overgrowth Accelerated atrophy after adolescence	10–15% volume loss in chronic MDD Reduced neurogenesis	Impaired stress buffering	ASD: Neurodevelopmental origin MDD: Stress-induced plasticity loss	[53]
Default Mode Network (DMN)	Overconnectivity in the posterior cingulate Linked to social cognition deficits	Hyperconnectivity in sgACC Correlates with rumination	Aberrant self-referential processing	ASD: Reduced anti correlation with ECN MDD: Increased sgACC PCC coupling	[49]
Cerebellum (Crus I/II)	Reduced Vermis VI VII volume Altered connectivity with PFC	7% smaller Crus I in MDD Predicts poor SSRI response	Disrupted cortico cerebellar loops	ASD: Early Purkinje cell loss MDD: Late onset degeneration	[50]

### 3.7. HPA Axis and Inflammation: Stress Response and Immune System Dysfunction

Neuroinflammation and the hypothalamic–pituitary–adrenal (HPA) axis are significant, frequently overlapping neurobiological processes linked to both ASD and MDD, and they are mostly to blame for their high comorbidity and comorbid vulnerabilities [19] (Figure 1). The body’s major neuroendocrine stress response mechanism, the HPA axis, integrates the release of cortisol, the main glucocorticoid in humans, in response to psychological or physical stressors [54,55]. It requires a careful balance between negative feedback for homeostasis and diurnal regularity to work properly [54,56]. In MDD, chronic stress causes hyperactivity of the HPA axis, which raises cortisol levels and causes an aberrant awakening response. The body’s negative feedback system is eventually weakened by this continuous activation, which keeps stress hormones from going back to normal [53]. Sustained high cortisol levels worsen depressive symptoms by damaging the hippocampus and PFC. The cycle of depression is exacerbated by the weakening of these two brain regions, which are essential for controlling mood [53]. Similar to this, aberrant HPA axis activity has been shown in ASD, though the patterns vary [57]. In both disorders, the hyper- or hypo-responsivity of stress response systems has significant effects on psychological functioning, anxiety, and emotional regulation [57].

Beyond the HPA axis, other research indicates that neuroinflammation and more widespread immune system abnormalities play a key role in the pathogenesis of both depression and ASD [58,59]. Neuroinflammation is inflammation that occurs in the brain and is typically caused by activated glial cells (microglia and astrocytes) and the release of chemokines and pro-inflammatory cytokines (such as tumor necrosis factor alpha (TNF-α), Interleukin 6 (IL-6), and interleukin 1 beta (IL-1β)) [58,60,61]. Evidence of chronic low-grade neuroinflammation, such as elevated microglial activation and pro-inflammatory cytokine levels in postmortem brain tissue, cerebrospinal fluid, and peripheral blood, has been widely reported in ASD [62]. The fundamental social communication dysfunction and repetitive behaviors that define autism are thought to be caused by this pro-inflammatory environment, which interferes with important neurodevelopmental processes such as synaptic pruning, neural plasticity, and the excitatory/inhibitory balance [63]. An important environmental risk factor for ASD is maternal immune activation during pregnancy, which is frequently brought on by an illness in the mother [64]. It causes oxidative stress and inflammation in the fetal brain, which can result in neurodevelopmental abnormalities [64]. Increased pro-inflammatory cytokines are seen in depressed individuals with MDD, and these inflammatory markers are further correlated with the severity of symptoms and the relative resistance to treatment [65]. Neuroinflammation is another characteristic of MDD that is increasingly recognized [65]. Chronic stress, which typically occurs before depressive illness, can trigger inflammatory cascades that damage cells and worsen depressive illness by triggering pathways such as oxidative stress [17,18]. The overall theory of immune brain axis dysregulation, in which stress, inflammation, and neurodevelopmental/mood problems are combined, is a potent unifying notion, even though the precise immune cell subsets or cytokine signatures may differ slightly between MDD and ASD [66]. To develop innovative treatment approaches, such as anti-inflammatory therapy or stress-management therapy, that may alleviate symptoms and improve the prognosis of patients with this somatotemporal comorbidity of autism and depression, it is imperative to clarify these neuroinflammatory and HPA axis dysregulations that are shared by the two conditions.

## 4. The Role of Alexithymia: Distinct Mechanisms of Emotional Processing

The comorbidity of MDD and ASD is significantly but subtly influenced by alexithymia, a personality construct marked by difficulty identifying and describing one’s own emotions, difficulty differentiating between feelings and somatic sensations of emotional arousal, and a limited imaginative life frequently accompanied by an externally orientated cognitive style [67]. Although alexithymia is not a diagnostic criterion for either condition, it is surprisingly common in both, significantly affecting emotional processing and complicating clinical presentation and treatment [68]. According to estimates, almost 50% of people with ASD have alexithymia, which is a far higher incidence than in the general population and frequently occurs regardless of intellectual capacity or the intensity of ASD symptoms [69]. Atypical connections in brain regions essential to emotional perception, such as the insula and anterior cingulate cortex (ACC), and neurodevelopmental deviations in interoception, the capacity to experience internal bodily states, may result in alexithymia in autistic individuals [70]. This also creates a gap between their internal emotional experience and their capacity to articulate it or put it into everyday language, making it difficult for them to identify internal signs of distress, such as those that typically appear in depression [70]. Therefore, rather than sadness or hopelessness, depression in autistic and alexithymic people may show up as behavioral changes such as increased repetitive behavior, irritation, aggression, loss of special interests, or greater physical problems [5]. This unusual presentation frequently results in “diagnostic overshadowing,” whereby depression symptoms are mistakenly ascribed to autism alone, delaying a proper diagnosis and course of treatment [5].

Alexithymia is another prevalent co-occurring symptom of MDD that affects a significant portion of the population [71]. In this instance, alexithymia is a contributing factor to depression as well as a risk factor. It may interfere with adaptive emotion regulation systems, making it more difficult to process and control negative feelings [71]. Although the pathophysiology of alexithymia may be different from that of ASD, the symptoms of depression may share the same difficulties with identifying and describing emotions [67]. Instead of a basic neurodevelopmental variation in emotional processing pathways, alexithymia in depression may be more the result of chronic affective distress or a pathological adjustment strategy created in reaction to an excessively stressful affective event [72].

Effective mental health treatment for depressed adults with ASD is hampered by the independent mechanisms of emotional processing, especially when viewed through the lens of alexithymia. When typical psychological procedures, such as CBT, call for verbal affect labelling or reflective evaluation of unavailable interior states, autistic people with alexithymia may find it difficult to participate [73]. This calls for assistance that emphasizes behavioral manifestations of discomfort, education on emotional concepts, and interoceptive development activities [73]. For instance, to promote emotional awareness and expression, interventions must make use of visual aids, literal communication, and specific interests [74]. Additionally, clinicians need to be extremely aware that people with autism, particularly those who are high on alexithymia, may not necessarily have depression just because they do not exhibit typical depressed affect [75]. Rather, habitual alterations, heightened sensitivity to stimuli, loss of previously learnt skills, or increased self-stimulatory behaviors might be used as main markers of underlying depressive moods [76]. To improve the mental health outcomes and quality of life for autistic adults who must manage the complicated world of comorbid depression, it is crucial to take into account alexithymia as both a general symptom and a distinct emotional processing mechanism [77].

## 5. Environmental and Psychological Factors: The Unique Role of Social Stress

Among environmental factors driving 3–4× higher depression rates in autism versus neurotypicals, social stress is particularly prevalent (65% co-occurring mental health diagnoses). Autistic individuals face chronic challenges in social interaction, communication, and sensory processing within neurotypical environments (90% sensory processing differences) [1,78]. This cumulative social stress manifests as camouflaging (71% prevalence, OR = 2.8 for depression), exhaustion (r = 0.62 with mental health decline), and early/lifelong rejection/bullying (66% lifetime prevalence), creating sustained depression risk distinct from episodic social stressors in the general population [79,80,81,82]. Sensory sensitivities (78%) and executive dysfunction precipitate autistic burnout (66% prevalence; precedes depression in 40% of cases). Continuous neurotype-environment mismatch, exacerbated by societal misunderstanding, necessitates neurodiversity-affirming interventions reducing depressive symptoms by 28% [83,84]. Therefore, rather than focusing only on individual-level interventions, the treatment of depression in autistic patients necessitates a well-established understanding of these particular environmental and psychological phenomena that embrace neurodiversity, create inclusive environments, and create support structures that lessen the burden of social masking and isolation [85].

## 6. Depression in Autistic Adults: Epidemiology and Unique Risk Factors

Although ASD has historically been studied primarily in pediatric populations, increasing attention has been directed toward the mental health challenges faced by autistic adults, among which depression represents one of the most prevalent and clinically significant comorbidities [86].

### 6.1. Epidemiological Findings

Epidemiological studies consistently indicate that autistic adults experience markedly higher rates of depressive disorders compared with the general population. Pooled prevalence estimates from meta-analyses range from 23% to 37% for current major depressive disorder (MDD) and exceed 40% for lifetime depression, representing approximately a 3- to 4-fold increase relative to neurotypical adults [87,88,89]. Emerging evidence indicates significant gender disparities in depression prevalence among autistic adults. Autistic females report higher rates of depressive symptoms and diagnosed MDD compared with autistic males, with odds ratios ranging from 1.5 to 2.5 depending on the study [90,91]. Several explanations have been proposed: autistic females may exhibit greater social camouflaging efforts, leading to chronic stress and delayed diagnosis; alternatively, diagnostic bias may underestimate depression in autistic males due to atypical presentation (e.g., externalizing behaviors rather than sadness). Importantly, transgender and gender-diverse autistic individuals show even higher depression rates, although research remains limited [92].

### 6.2. Adult-Specific Risk Factors

#### 6.2.1. Late Diagnosis

A substantial proportion of autistic adults, especially those with average or high cognitive abilities, receive their diagnosis only in adulthood after years of unrecognized difficulties [87,88]. Late diagnosis is associated with significantly poorer mental health outcomes. A longitudinal cohort study found that autistic adults diagnosed after age 21 had 2.3-fold higher odds of lifetime depression compared with those diagnosed in childhood, even after controlling for autism symptom severity [93]. The proposed mechanism involves a prolonged period of “self-blindness” and internalized stigma: individuals attribute their social failures, sensory overload, and executive dysfunction to personal flaws rather than a neurodevelopmental condition, leading to chronic shame, low self-esteem, and depressive rumination [87,88]. Late-diagnosed adults also report fewer years of appropriate accommodations and support, compounding cumulative stress.

#### 6.2.2. Social Camouflaging (Masking)

Many autistic adults consciously suppress or modify their autistic traits to conform to neurotypical social expectations. While camouflaging may facilitate short-term social acceptance, sustained masking is associated with significant psychological strain, emotional exhaustion, identity confusion, and an increased risk of depressive symptoms and autistic burnout [80,94]. A large survey study found that higher camouflaging scores predicted depression severity independently of core autism symptom severity, suggesting that the effort of masking is itself a unique risk factor [94].

#### 6.2.3. Structural Barriers

Autistic adults frequently encounter barriers in employment (underemployment, workplace sensory and social challenges), independent living (difficulty accessing housing and daily living supports), and social relationships (loneliness, reduced social networks). These structural factors contribute to persistent loneliness, financial strain, and reduced quality of life, each independently associated with depression in longitudinal studies [87,88].

#### 6.2.4. Interaction with Neurobiological Vulnerabilities

The psychosocial risk factors described above do not operate in isolation. Chronic stress from late diagnosis, masking, and structural barriers can exacerbate underlying neurobiological vulnerabilities, creating a self-reinforcing loop that lowers the threshold for depressive episodes [19,80].

### 6.3. Clinical Implications

Depressive symptoms in autistic adults often present atypically manifesting as increased irritability, loss of special interests, or behavioral regression rather than sad mood and can be difficult to recognize due to overlapping features such as social withdrawal, reduced motivation, or alexithymia [88]. Consequently, depression may be underdiagnosed or inadequately treated in this population. Recognizing the distinct epidemiological patterns (including gender differences) and the specific risk factors outlined above is essential for improving diagnostic accuracy, guiding targeted interventions, and informing the development of supportive clinical and social care strategies for autistic adults.

## 7. Diagnostic Challenges: Recognizing Depression in Autistic Individuals

Diagnosing depression in autistic adults is challenging due to symptom overlap with ASD features (social withdrawal, routine disruption, sleep irregularities), often leading to delayed intervention [1] (Figure 2). Clinicians frequently attribute these symptoms solely to autism, missing co-occurring mood disorders [2].

Standard self-report depression assessments are unreliable for autistic individuals due to atypical communication styles and limited emotional expression vocabulary [95]. These tools, designed for neurotypical populations, fail to capture internal depressive states [96].

Autistic people may display more irritability, aggression, self-harming behaviors (such as head banging or biting), a seeming regression of previously learnt skills (such as communication or self-care), increased sensory sensitivity, or a shift in the focus or intensity of their special interests (such as a favorite interest turning into an obsessive or morbid one) in place of the stereotypical depressive behaviors [97]. These behaviors, whether classified as “challenging behaviors” or as characteristics of their autism, are most suggestive of deeper-seated depression. These difficulties are exacerbated by the absence of standardized, validated tests created especially for the many communication styles and unique ways that depression manifests in the autistic community [97]. Many cases of depression in autistic people go undetected and untreated due to this diagnostic gap, which lowers their quality of life, increases their functional impairment, and increases their risk of suicide [98]. To detect depression in adults with autism, clinical practice must adopt a more individualized approach based on careful behavioral observation, collateral history from caregivers or family members, and autism-informed assessment methods [99]. In practice, this may include clinician-administered interviews, multi-informant assessment, autism-informed clinical observation, and the adapted use of common depression scales (e.g., the PHQ-9 or Beck Depression Inventory) with concrete language support, visual prompts, and structured clarification to improve symptom reporting in individuals with alexithymia or communication differences [95,99].

## 8. Therapeutic Strategies

The strength of evidence across therapeutic strategies is uneven. Robust efficacy data are derived mainly from general major depressive disorder populations, whereas ASD-specific evidence remains limited, heterogeneous, and often based on small trials or secondary outcomes. The discussion below therefore prioritizes clinical relevance, safety, and the distinction between evidence derived from neurotypical versus autistic samples.

### 8.1. Selective Serotonin Reuptake Inhibitors

**Figure 3:**
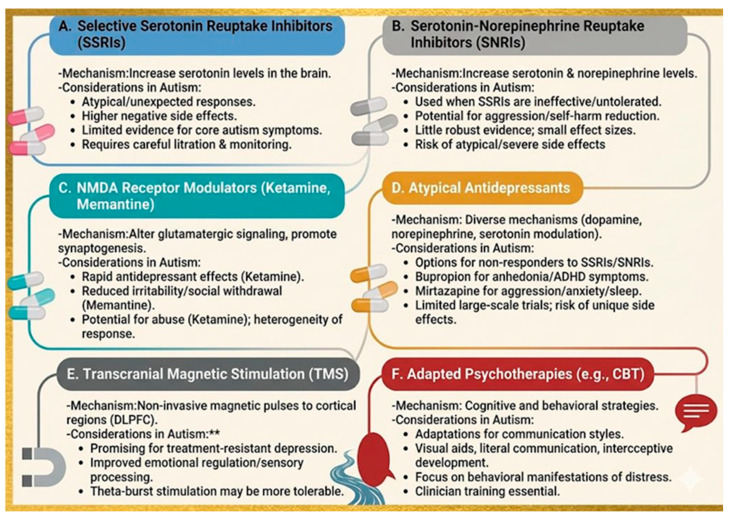
Therapeutic Strategies for Depression in Autism Spectrum Disorder (ASD). This figure outlines various pharmacological and non-pharmacological interventions used to treat depression in autistic individuals. It details the mechanism of action for each strategy, including Selective Serotonin Reuptake Inhibitors (SSRIs), Serotonin Norepinephrine Reuptake Inhibitors (SNRIs), N-methyl-D-aspartate (NMDA) Receptor Modulators (Ketamine, Memantine), Atypical Antidepressants, and Transcranial Magnetic Stimulation (TMS). Crucially, the figure highlights specific considerations for each therapy in the context of autism, such as atypical responses, potential side effects, and the need for careful titration and tailored approaches. It also includes Adapted Psychotherapies like Cognitive Behavioral Therapy (CBT), emphasizing modifications for communication and sensory differences. Note: “**” indicates considerations specific to autistic individuals, including variability in treatment response and sensory sensitivity.

SSRIs remain first-line pharmacotherapy for major depressive disorder in the general population; however, their use in autistic individuals requires greater caution because efficacy and tolerability data are more limited and less consistent in ASD-specific studies [41,100,101,102,103]. Available evidence suggests that some autistic adults may benefit, particularly when depression or anxiety is clearly present, but response is heterogeneous and the evidence base remains substantially weaker than in neurotypical depression [41,101,102]. Importantly, most robust data on antidepressant efficacy come from non-autistic populations, whereas ASD-specific evidence is derived mainly from smaller trials, observational reports, or studies focused on anxiety, irritability, or repetitive behaviors rather than depression alone [41,101,102] (Figure 3).

Autistic individuals may also be more vulnerable to adverse effects, including behavioral activation, irritability, agitation, sleep disturbance, and, in rare cases, manic symptoms, underscoring the need for low starting doses, slow titration, and close monitoring [101,102]. Overall, SSRIs should be considered a cautious, individualized option rather than a universally effective treatment for ASD–depression comorbidity, and treatment decisions should weigh potential benefit against side-effect burden and the limited ASD-specific evidence base. A comparative summary of therapeutic approaches relative to SSRIs is provided in Table 3.

### 8.2. Serotonin Norepinephrine Reuptake Inhibitors

Another class of antidepressants commonly used to treat MDD in the general population is serotonin norepinephrine reuptake inhibitors (SNRIs) [104] (Figure 3). These drugs, which work by preventing the brain from reabsorbing serotonin and norepinephrine, increase their availability in the synaptic cleft, thereby improving mood, alertness, and attention [104]. As evidence of their wide range of therapeutic uses, SNRIs are given for ailments other than MDD, such as fibromyalgia in neurotypical people, generalized anxiety disorder, and specific chronic pain syndromes [105]. SNRIs are viewed as a possible pharmacological intervention in the context of ASD, where depression and anxiety are highly comorbid [106]. This is especially true when SSRIs are ineffective or poorly tolerated, or when symptoms such as significant fatigue, anhedonia, or specific behavioral challenges (such as aggression, impulsivity, or self-harming behaviors) are prevalent [106]. The documented imbalance of the serotonergic and noradrenergic systems in autism, which is linked to several autistic symptoms and co-occurring conditions, is the theoretical basis for the attraction of SNRIs in ASD [107]. However, there is still little and frequently conflicting data to support the widespread effectiveness of SNRIs for treating depression and related symptoms in autistic people [108]. Robust large-scale randomized controlled trials are rare, despite some clinical observations and smaller studies suggesting that some SNRIs, like venlafaxine, may be beneficial for specific behaviors like aggression or self-harming behaviors, often at doses lower than those typically prescribed for depression, or milnacipran for improving impulsivity and social functioning by reducing inattention [109]. Meta-analyses of antidepressants in ASD typically conclude that, although there may be slight improvements in global symptoms or restricted and repetitive behaviors, the effect sizes are small, and it is challenging to draw firm conclusions about their efficacy for core autism symptoms or even depression because of methodological flaws and inconsistent results from different studies [110]. Since autistic people are more likely than neurotypical groups to experience atypical or severe side effects and are frequently more sensitive to the effects of medications, this is an important factor to take into account when prescribing SNRIs to them [111]. For autistic people who may already have sensory sensitivity, rigid routines, or trouble expressing their inner states, SNRI side effects such as gastrointestinal problems, headaches, dizziness, sweating, sleep disturbances, changes in appetite or weight, and emotional blunting can be especially difficult [111]. A highly customized approach, careful titration, and close monitoring by medical professionals are required due to the possibility of increased agitation, hyperactivity, or even uncommon but serious adverse events like serotonin syndrome [111]. Furthermore, the expected therapeutic effects observed in neurotypical individuals may not translate directly due to the underlying neurobiological differences in how autistic brains process neurotransmitters [111]. This highlights the urgent need for more focused research to establish clear guidelines for the use of SNRIs in this diverse population as summarized in Table 3.

### 8.3. Atypical Antidepressants

Since they work through a variety of mechanisms involving distinct neurotransmitter systems, such as dopamine, norepinephrine, and serotonin, and frequently have distinct receptor profiles, atypical antidepressants are a broad class of drugs that do not neatly fall into the categories of SSRIs or SNRIs [112] (Figure 3). Drugs with different pharmacological effects and adverse effect profiles, such as bupropion, mirtazapine, trazodone, vilazodone, and vortioxetine, are included in this family [112]. When people do not respond to or tolerate first-line SSRIs/SNRIs, or when certain symptoms such as severe fatigue (bupropion), insomnia, and appetite changes (mirtazapine, trazodone), or cognitive dysfunction (vortioxetine) are prevalent, atypical antidepressants are useful options for MDD in the general population [113].

Atypical antidepressants are being investigated more and more for autistic people who are depressed, particularly in light of their inconsistent and frequently difficult reactions to SSRIs and SNRIs [40]. Their use in autism is justified by the intricate neurological foundations of the disorder, which include dysregulation of several different neurotransmitter systems [40]. For instance, despite the paucity of clinical studies, bupropion, a norepinephrine dopamine reuptake inhibitor, has demonstrated some promise in treating depression and the symptoms of attention deficit/hyperactivity disorder (ADHD) in children with autism [114]. Because of its dopaminergic activity, it may be especially useful in treating anhedonia and motivational deficiencies that are frequently observed in both autism and depression [114]. Mirtazapine, an α2 adrenergic antagonist that blocks specific SERTs, is occasionally used off-label in autistic people, mainly for aggression, anxiety, and sleep disturbances [115]. Some open-label studies indicate that it has a moderately positive effect on these related symptoms, such as depression and irritability [115].

For autistic people who struggle with insomnia or weight loss linked to depression, its sedative qualities and potential for increased appetite may be helpful. Although it has antidepressant effects at larger dosages, trazodone, a serotonin antagonist and reuptake inhibitor, is primarily used at lower doses to treat insomnia in both neurotypical and autistic populations [116]. Its sedative effects can indirectly reduce depressive symptoms associated with severe sleep problems, although there is a dearth of data focused on its antidepressant efficacy in autistic individuals [116]. In patients with depression, including those with Alzheimer’s disease and depressive symptoms, vortioxetine, a multimodal antidepressant that directly controls 5-HT receptor activity and inhibits the 5-HT transporter, has demonstrated promise in enhancing mood and cognitive performance [117]. Although particular studies in the autistic population are still in their infancy, their distinct mode of action may be able to treat the cognitive inflexibility and emotional control issues frequently observed in autistic people with depression.

Like other psychotropics, atypical antidepressants in autism carry several important risks, including the possibility of distinct or more severe side effects [118]. Lower initial doses and slower titration may be necessary for autistic people since they may be more susceptible to the side effects of medications [118]. In a group already prone to neurological anomalies, bupropion, for example, presents a risk of seizures, psychosis, and mania, all of which would need close monitoring [119]. Increased appetite and temporary sedation are common side effects of mirtazapine that, although occasionally helpful, can also be problematic [120]. A substantial portion of current therapy is based on clinical experience and extrapolation from neurotypical populations because there are so few large-scale, placebo-controlled trials explicitly focused on atypical antidepressants for depression in autistic adults [40]. This demonstrates the urgent need for more thorough research to develop precise usage guidelines, optimize dosage plans, and gain a deeper understanding of their effects on the complex neurobiology and variety of depression presentations found in the autistic community as summarized in Table 3.

### 8.4. NMDA Receptor Modulators

With unique mechanisms and clinical characteristics, N-methyl-D-aspartate (NMDA) receptor modulators, ketamine and memantine in particular, are becoming more and more popular as targeted therapy for depression in people with autism [121] (Figure 3). By altering glutamatergic signaling, promoting synaptogenesis through the BDNF and mammalian target of rapamycin (mTOR) pathways, and reducing inflammatory markers associated with depression, ketamine, a noncompetitive NMDA receptor antagonist, produces rapid antidepressant effects [122]. A double-blind trial showed that memantine, a low-affinity NMDA receptor antagonist approved for Alzheimer’s disease, decreased irritability and social withdrawal in autistic children (dose: 2.5–20 mg/day) [123]. Glutamate normalization in prefrontal amygdala circuits and decreased excitotoxicity, especially in those with GRIN2B mutations (a genetic subtype associated with autism), are two suggested mechanisms [124]. Memantine’s tolerability in autism is supported by real-world data (with weak side effects such as fatigue and dizziness) [125]. One of the challenges is heterogeneity: memantine’s benefits are more noticeable in people with associated ADHD or sensory hypersensitivity, while ketamine’s effectiveness may be stronger in autistic patients with elevated inflammatory markers (IL-6, TNF-α) [126,127]. Leveraging memantine’s neuroprotective properties and ketamine’s rapid onset, current trials are investigating ketamine–memantine combinations. Despite its potential, both medications need strict autism-specific criteria, particularly for long-term usage, considering the potential for abuse of ketamine and the unknown consequences of memantine on young people’s neurodevelopment [128] as summarized in Table 3.

### 8.5. Transcranial Magnetic Stimulation

To modify neural activity in treatment-resistant depression (TRD), transcranial magnetic stimulation (TMS), a non-invasive brain stimulation method, employs electromagnetic coils to deliver targeted magnetic pulses to specific cortical regions, most commonly the dorsolateral prefrontal cortex (DLPFC) [129] (Figure 3). The FDA approved TRD in 2008 after repeated TMS (rTMS) demonstrated strong efficacy in neurotypical populations, with response rates of 50–60% and remission rates of 30–35% among patients resistant to treatment [130]. Frequency–dependent modulation of cortical excitability is the therapeutic mechanism: low frequency stimulation (1 Hz) inhibits the hyperactive right DLPFC, whereas high-frequency stimulation (10 Hz) increases activity in the hypoactive left DLPFC, thereby rebalancing the front-limbic circuits involved in mood regulation [131]. Although there is still little data, new research points to a possible application for depressed autistic people. Similar response rates in adults with autism were found by an open-label trial conducted in 2020 [132]. Other advantages were noted in emotional regulation and sensory processing, which may have been mediated by secondary effects on connectivity within the salience and default mode networks [133]. Due to its shorter session time (35 min compared with 37 min for standard trims), theta-burst stimulation (TBS), an accelerated procedure that delivers structured bursts at 5 Hz, shows particular promise and may be more tolerable for people with sensory sensitivity [134]. The necessity for customized coil placement (since structural variations in prefrontal cortex morphology may affect targeting), cautious handling of sensory discomfort from clicking noises and scalp sensations, and possible variations in neuroplasticity responses are among the difficulties unique to autism [134]. According to current therapeutic guidelines, normal depression protocols (10 Hz left DLPFC or intermittent TBS) should be used while keeping an eye out for unusual reactions [135]. The long-term effects of TMS on autistic neurobiology remain unknown, particularly regarding its interaction with atypical cortical excitation-inhibition balance, despite its generally safe profile (with a seizure risk of less than 0.1%) [132] as summarized in Table 3.

## 9. Natural Alternatives and Microbial-Based Interventions

### 9.1. Plant-Derived Compounds

Many of these interventions converge on shared biological pathways, including modulation of neuroinflammation, enhancement of BDNF signaling, and regulation of the gut–brain axis; however, a substantial proportion of the supporting evidence is derived from preclinical studies, and its direct clinical applicability should therefore be interpreted with caution.

#### 9.1.1. *Curcuma longa* (Curcumin)

Curcumin has attracted interest because of its anti-inflammatory and antioxidant actions, and several preclinical studies suggest possible antidepressant and neuroprotective effects [136,137,138,139,140]. However, most of this evidence is derived from animal or mechanistic studies rather than ASD-specific clinical trials. Available human data support curcumin mainly as a potential adjunct in depression-related inflammatory states, not as an established treatment for ASD–depression comorbidity. Accordingly, curcumin should be viewed as a promising but still preliminary adjunctive strategy pending better ASD-specific clinical evidence.

#### 9.1.2. *Hypericum perforatum* (St. John’s Wort)

St. John’s Wort is one of the best-studied plant-based interventions for mild to moderate depression in the general population, where its efficacy may be comparable to SSRIs in some studies [141,142,143,144,145]. However, ASD-specific evidence remains very limited, and its clinical use in autistic individuals should be approached cautiously. Its main practical limitation is the high potential for herb–drug interactions, especially through cytochrome P450 induction, which can alter the effectiveness of many commonly used medications [141,143]. Thus, St. John’s Wort may be discussed as a potential option in general depression, but not as an established ASD-specific intervention.

#### 9.1.3. *Ginkgo biloba* 

*Ginkgo biloba* is among the most widely used natural cognitive enhancers and has demonstrated promising neuroprotective and antidepressant effects. The standardized extract EGb 761, rich in flavonoids and terpenoids (notably ginkgolides and bilobalide), exhibits potent antioxidant and free radical scavenging activity, mitigating neuronal oxidative damage and excitotoxicity [146,147]. Mechanistically, bilobalide and ginkgolides modulate neurotransmission by enhancing dopaminergic and serotonergic signaling, while simultaneously reducing glutamatergic excitotoxicity and regulating GABAergic tone [148,149]. These combined effects contribute to improved synaptic plasticity, cognitive flexibility, and emotional regulation.

Experimental models demonstrate that Ginkgo supplementation alleviates depressive-like behaviors and supports neuronal function [146]. Clinically, randomized controlled trials have reported that EGb 761 improves mood disturbances, cognitive rigidity, and behavioral symptoms in dementia and depressive disorders [147]. Importantly, meta-analyses suggest that *Ginkgo biloba* extract provides modest but consistent improvements in cognitive function and mood, particularly in patients with mild cognitive impairment or neuropsychiatric comorbidities [149]. Collectively, these findings support the role of *Ginkgo biloba* as a potential adjunctive therapy through multimodal neurotransmitter modulation and neuroprotective actions.

#### 9.1.4. Polyphenols (Resveratrol, Catechins, Quercetin, EGCG)

Many of these interventions converge on shared biological pathways, including modulation of neuroinflammation, enhancement of BDNF signaling, and regulation of the gut–brain axis.

Polyphenols, a diverse group of plant-derived compounds including resveratrol, catechins, quercetin, and epigallocatechin gallate (EGCG), exert potent antioxidant effects that directly impact brain function and plasticity. These compounds scavenge reactive oxygen species and upregulate endogenous antioxidant defenses such as superoxide dismutase and glutathione peroxidase, thereby reducing oxidative stress, a critical driver of neurodegeneration [150]. Significantly, polyphenols modulate hippocampal BDNF expression through phosphorylation of cAMP response element binding protein (CREB) and epigenetic remodeling, mechanisms that enhance neuronal survival and synaptic plasticity [151].

Resveratrol has been shown to activate sirtuin 1 (SIRT1) and PI3K/Akt signaling, thereby upregulating BDNF and promoting synaptic resilience, while catechins such as EGCG reduce NF-κB-driven inflammation and facilitate neurogenesis [152]. Preclinical studies consistently demonstrate that polyphenols ameliorate cognitive decline and depressive-like behaviors by improving hippocampal plasticity and neurogenesis [151,153]. Clinical trials and epidemiological data further confirm that polyphenol-rich diets are associated with improved neurocognitive outcomes and protection against age-related neurodegeneration [150]. Collectively, these findings highlight polyphenols as promising natural agents that act via BDNF modulation and synaptic support, offering therapeutic potential for depression and neurodegenerative disorders.

### 9.2. Microbial-Based Interventions

#### 9.2.1. Probiotics (Lactobacillus and Bifidobacterium)

Probiotics have been investigated as microbiota–gut–brain interventions that may influence emotional regulation, gastrointestinal symptoms, and stress-related biology [154,155,156]. Preclinical studies suggest possible effects on tryptophan metabolism, serotonergic signaling, and HPA axis regulation, but these mechanistic findings should not be overinterpreted clinically. At present, probiotics should be considered promising adjunctive strategies rather than established treatments for ASD–depression comorbidity.

#### 9.2.2. Clinical Evidence of Probiotics in ASD

Clinical evidence indicates that probiotic supplementation can alleviate not only core neuropsychiatric symptoms but also gastrointestinal comorbidities commonly observed in individuals with ASD. Randomized controlled trials (RCTs) involving *Lactobacillus plantarum* PS128 demonstrated reductions in anxiety and improvements in behavioral scores, including social responsiveness and hyperactivity, in children and adolescents with ASD [157,158]. Similarly, supplementation with *Bifidobacterium animalis* subsp. Lactis BPL1 showed modest but significant reductions in withdrawal/depressive symptoms in pediatric populations with neurodevelopmental disorders [159].

Systematic reviews further support these findings, highlighting that probiotics—particularly strains of Lactobacillus and Bifidobacterium improve depressive and anxiety symptoms in children with ASD, while prebiotics alone appear less effective [157]. In parallel, a recent review emphasized that *Lactiplantibacillus plantarum* strains exert beneficial effects on both gastrointestinal integrity and behavioral symptoms, with PS128 showing consistent improvements in sociability, anxiety, and cognition in preclinical and clinical models [160]. Mechanistically, probiotics exert effects through modulation of gut–brain signaling and neuroimmune pathways [161].

Taken together, these clinical studies suggest that probiotic supplementation reduces anxiety, depressive symptoms, and gastrointestinal comorbidities in ASD, particularly when well-characterized strains such as *L. plantarum* PS128 and *Bifidobacterium* spp. are administered under controlled regimens.

However, findings should be interpreted with caution due to the heterogeneity of microbial strains, variability in study designs, and differences in population characteristics, particularly within ASD cohorts.

#### 9.2.3. Clinical Trials of Probiotics in ASD

Clinical studies in ASD suggest that selected probiotic strains may improve gastrointestinal symptoms and some behavioral outcomes, with a few studies also reporting reductions in anxiety or depressive symptoms [157,158,159,160]. However, the literature remains heterogeneous with respect to strain selection, dose, treatment duration, age group, and outcome measures. Moreover, most trials are small and were not designed primarily to evaluate major depressive disorder in autistic populations. Therefore, current evidence supports cautious optimism, but not firm conclusions regarding antidepressant efficacy in ASD [157,158,159,160,161].

#### 9.2.4. Postbiotics (Short-Chain Fatty Acids and Microbial Metabolites)

Postbiotics, including short-chain fatty acids and other microbial metabolites, are increasingly discussed as modulators of neuroimmune and gut–brain signaling [162,163,164,165,166]. Their proposed relevance to depression is supported mainly by mechanistic and preclinical data, whereas direct clinical evidence in ASD–depression comorbidity is still limited. For this reason, postbiotics should currently be framed as experimental or hypothesis-generating adjuncts rather than clinically established interventions. Future work should determine whether specific postbiotic profiles have reproducible therapeutic relevance in autistic populations.

### 9.3. Toward Integrated Treatment Strategies for ASD–Depression Comorbidity

#### 9.3.1. Adapted Psychotherapeutic Interventions

Psychotherapeutic approaches remain a cornerstone in the management of depression; however, conventional therapies often require modification when applied to autistic individuals [167]. CBT, which is widely used for depressive disorders, may need to be adapted to accommodate the cognitive and communication profiles frequently observed in ASD [167]. For example, therapeutic strategies that incorporate visual supports, structured sessions, concrete language, and explicit emotion-recognition training have been shown to improve engagement and treatment outcomes among autistic patients [167]. In addition, therapists may need to focus more explicitly on developing emotional awareness and coping strategies for managing social stressors, as many autistic individuals experience difficulties identifying and articulating emotional states due to alexithymia [167]. Tailoring psychotherapeutic techniques to the neurocognitive characteristics of ASD can therefore enhance the effectiveness of depression treatment while promoting greater patient participation and therapeutic alliance.

#### 9.3.2. Social and Environmental Support Interventions

Beyond individual psychotherapy, addressing the broader psychosocial context is critical for managing depression in autistic individuals [168]. Autistic adults frequently face persistent challenges related to employment, social relationships, and independent living, all of which can contribute to chronic stress and depressive symptoms. Interventions aimed at improving social inclusion, such as peer support programs, autism-informed counseling services, and supported employment initiatives, have demonstrated potential benefits for psychological well-being [168]. Programs that foster community integration and reduce social isolation may also mitigate some of the environmental stressors that contribute to depressive vulnerability [168]. Importantly, interventions that promote neurodiversity-affirming environments, rather than attempting to normalize autistic traits, may reduce the psychological burden associated with social masking and improve long-term mental health outcomes.

#### 9.3.3. Precision Pharmacotherapy

Pharmacological treatment of depression in autistic individuals presents unique challenges. While SSRIs and other antidepressants are commonly prescribed, evidence regarding their efficacy and tolerability in autistic populations remains limited and sometimes inconsistent [41]. Autistic individuals may exhibit altered sensitivity to psychotropic medications, potentially due to differences in neurochemical signaling or pharmacokinetic profiles. Consequently, careful dose titration, close monitoring of side effects, and individualized treatment planning are often necessary [41]. Emerging research also suggests that targeting neuroinflammatory pathways, glutamatergic signaling, or mitochondrial dysfunction may be promising therapeutic directions, particularly in cases where conventional antidepressants are less effective [52,169].

#### 9.3.4. Multimodal and Personalized Care Models

Given the multifactorial nature of ASD–depression comorbidity, multimodal treatment frameworks have been proposed and increasingly adopted as pharmacological, psychological, and social interventions [170]. Such approaches recognize that depression in autistic individuals may arise from a complex interplay between biological vulnerability and environmental stressors [170]. Integrated care models that combine adapted psychotherapy, individualized pharmacotherapy, and social support interventions may therefore provide the most effective strategy for improving mental health outcomes [171]. Multidisciplinary collaboration among psychiatrists, psychologists, neurologists, and social care professionals is particularly important in developing personalized treatment plans that address both the neurodevelopmental features of ASD and the affective symptoms of depression [172]. By adopting a comprehensive and individualized approach, clinicians may be better positioned to address the unique clinical challenges associated with ASD–depression comorbidity across the lifespan [172].

A summary of currently investigated pharmacological and psychosocial interventions for ASD–depression comorbidity, along with their level of clinical evidence, is presented in Table 3.

#### 9.3.5. Mechanistic Stratification and Targeted Intervention Hypotheses

The evidence reviewed across genetic overlap [14,15,16,17,18,19,20,21], neurotransmitter dysregulation [22,23,24,25,26,27,28,29,30,31,32,33,34,35], HPA axis and inflammatory alterations [48,49,50,51,52,53,54,55,56,57,58,59,60,61,62,63], neuromodulation studies [108,109,110,111,112,113,114,115,116,117,118,119,120,121,122], and plant and microbiota-based interventions [123,124,125,126,127,128,129,130,131,132,133,134,135,136,137,138,139,140,141,142,143,144,145,146,147,148] suggests that ASD–depression comorbidity is biologically heterogeneous. Rather than representing a uniform clinical entity, depressive symptoms in autistic individuals may emerge from distinct but overlapping mechanistic pathways.

Based on the convergent mechanisms summarized in this review, we propose a conceptual mechanistic stratification framework that links dominant biological profiles to targeted intervention hypotheses. This framework is intended to integrate neurobiological, environmental, and translational therapeutic evidence discussed above.

The proposed mechanistic stratification framework and corresponding targeted intervention hypotheses are summarized in Table 4.

This proposed mechanistic stratification framework integrates the convergent neurobiological pathways summarized in Figure 1 and the shared genetic architecture presented in Table 1. The evidence supporting each subtype is derived from studies discussed throughout this review, including genetic overlap [14,15,16,17,18,19,20,21], neurotransmitter dysregulation [22,23,24,25,26,27,28,29,30,31,32,33,34,35], immune and HPA axis alterations [48,49,50,51,52,53,54,55,56,57,58,59,60,61,62,63], neuromodulation trials [116,117,118,119,120,121,122], and plant and microbiota-based interventions [123,124,125,126,127,128,129,130,131,132,133,134,135,136,137,138,139,140,141,142,143,144,145,146,147,148].

While conceptual in nature, this framework highlights the potential value of biomarker-informed and individualized treatment strategies in ASD–depression comorbidity. Future longitudinal studies and biomarker-stratified clinical trials in autistic populations are necessary to determine whether distinct mechanistic profiles predict differential therapeutic response.

Biomarker variability represents a major challenge in ASD–depression research. Inter-individual differences have been reported across neuroinflammatory markers, HPA axis measures, and neuroimaging findings, likely reflecting variation in polygenic risk, developmental stage, psychosocial stress exposure, comorbidities, and timing of assessment [78,173,174,175]. These sources of heterogeneity suggest that single biomarkers are unlikely to be sufficient for clinical stratification. Instead, multimodal panels integrating inflammatory, endocrine, imaging, and behavioral measures may provide a more reliable framework for subgroup identification. Early machine-learning studies support this direction, but these approaches remain preliminary and require prospective validation in autistic populations before clinical application [175]. A comparative overview of therapeutic strategies relative to SSRIs is presented in Table 5.

## 10. Future Perspectives and Limitations

One of the main limitations of this review is the heterogeneity of research designs and the small sample sizes characteristic of much autism-depression comorbidity research. Several studies employ self-report measures, which are challenging for autistic individuals due to communication impairment and alexithymia, and thus may distort diagnostic and symptom severity data. Furthermore, reliance on a few animal models, particularly in gut–brain axis research, limits the generalizability of findings to the complex human neurodevelopmental context. There is also a need for long-term longitudinal studies that are essential for tracing the developmental trajectory of depression in autism, as well as the long-term efficacy of therapeutic interventions.

It is a priority for future research to develop more objective, biologically based diagnostic tools, i.e., biomarkers, to augment clinical assessments. Greater emphasis on large, multisite clinical trials is needed to test and compare the effectiveness of different treatment approaches, both pharmacologic and non-pharmacologic, specifically in the autistic population. Additionally, future studies need to discuss the potential of novel treatments like psychedelics and neurofeedback that may offer new treatment possibilities. Lastly, the application of a participatory research model, in which autistic individuals are part of the study design and implementation, needs to be a critical element to help ensure that study questions and findings are relevant and translate to the community. By transcending these constraints, future research can pave the way for more accurate diagnoses and tailored interventions that are effective and actually improve the mental health and well-being of autistic individuals.

## 11. Limitations and Future Perspectives

### 11.1. Limitations

This review has several limitations. First, as a narrative review, it does not follow a formal systematic-review or meta-analytic design and is therefore potentially vulnerable to selection bias. Second, the evidence base is heterogeneous and includes both clinical and preclinical studies that differ in sample size, methods, and outcome measures. Third, many studies in ASD–depression research involve small samples and rely on self-report instruments that may be less accurate in autistic populations because of communication differences and alexithymia. Finally, several mechanistic and therapeutic interpretations remain hypothesis-generating and require validation in longitudinal studies and randomized controlled trials.

### 11.2. Future Perspectives

Future research should prioritize larger multisite and longitudinal studies to clarify developmental pathways, biomarkers, and treatment response in ASD–depression comorbidity. Greater emphasis is also needed on ASD-specific randomized controlled trials for pharmacological, neuromodulatory, psychosocial, and microbiota-targeted interventions. Emerging approaches, including neurofeedback, psychedelics, and biomarker-informed stratification, remain promising but should be interpreted cautiously until supported by stronger evidence. Participatory research that actively involves autistic individuals in study design and implementation will be essential for improving clinical relevance and neurodiversity-affirming care.

## 12. Conclusions

Depression in individuals with autism spectrum disorder represents a complex and multifactorial clinical challenge arising from the interaction of shared neurobiological mechanisms and cumulative psychosocial stressors. Converging evidence highlights the roles of neurotransmitter dysregulation, neuroinflammation, HPA axis alterations, and large-scale brain network disruptions, alongside environmental factors such as social stress and masking behaviors.

Current treatment approaches remain limited by heterogeneous responses and a lack of ASD-specific evidence. Emerging strategies, including glutamatergic modulators, neuromodulation techniques, plant-derived compounds, and microbiota-targeted interventions, show promise but require further validation.

Advancing the field will depend on integrating biological stratification with personalized, neurodiversity-informed care models. Multidisciplinary and multimodal approaches are essential to improve diagnostic accuracy, optimize treatment strategies, and enhance mental health outcomes in autistic individuals.

## Figures and Tables

**Table 3:** Current therapeutic strategies for ASD–depression comorbidity and their level of clinical evidence.

Therapeutic Approach	Mechanism/Target	Evidence in ASD	Evidence for Depression	Level of Clinical Evidence	References
Selective Serotonin Reuptake Inhibitors (SSRIs) (e.g., fluoxetine, sertraline)	Increase synaptic serotonin; modulate mood regulation circuits	Mixed results; sometimes used for anxiety and repetitive behaviors in ASD	Widely established first-line treatment for major depressive disorder	Moderate clinical evidence in ASD populations	[34,35,87,88,89,90]
Serotonin–Norepinephrine Reuptake Inhibitors (SNRIs) (e.g., venlafaxine, duloxetine)	Enhance serotonergic and noradrenergic neurotransmission	Limited ASD specific studies	Effective antidepressants in the general population	Limited ASD specific evidence	[91,92,93,94,95,96,97,98]
Atypical Antidepressants (e.g., bupropion, mirtazapine, trazodone, vortioxetine)	Dopamine and serotonin modulation; multimodal receptor effects	Limited and heterogeneous data in ASD	Established use in general MDD populations	Limited ASD specific evidence	[99,100,101,102,103,104,105,106,107]
NMDA Receptor Modulators (e.g., ketamine, memantine)	Modulate glutamatergic signaling and synaptic plasticity	Investigational use in ASD related symptoms	Rapid antidepressant effects in treatment-resistant depression	Experimental evidence	[108,109,110,111,112,113,114,115]
Transcranial Magnetic Stimulation (TMS)	Modulate cortical excitability and fronto-limbic circuits	Emerging evidence in ASD populations	FDA approved for treatment-resistant depression	Moderate evidence (MDD); limited ASD data	[116,117,118,119,120,121,122]
Curcumin (*Curcuma longa*)	Anti-inflammatory; NF-κB and Nrf2 modulation; BDNF upregulation	Preclinical and adjunctive evidence	Meta analytic support in depressive symptoms	Emerging translational evidence	[123,124,125,126,127]
*Hypericum perforatum* (St. John’s Wort)	Monoamine reuptake inhibition; TRPC6 activation	Limited ASD specific data	Comparable efficacy to SSRIs in mild–moderate depression	Moderate evidence (general depression)	[128,129,130,131,132]
*Ginkgo biloba*	Antioxidant; dopaminergic and serotonergic modulation	Limited ASD evidence	Adjunctive mood and cognitive benefits	Emerging evidence	[133,134,135,136]
Polyphenols (e.g., resveratrol, EGCG)	Antioxidant; BDNF modulation; anti-inflammatory effects	Preclinical ASD relevance	Evidence for mood and cognitive improvement	Emerging evidence	[137,138,139,140]
Probiotics (*Lactobacillus*, *Bifidobacterium*)	Modulate gut–brain axis; regulate serotonin and HPA axis	RCTs showing behavioral improvements in ASD	Meta analytic support for depressive symptom reduction	Emerging clinical evidence	[141,142,143,144,145,146,147,148]
Postbiotics (Short-chain fatty acids)	Epigenetic modulation; immune regulation; BBB stabilization	Preclinical ASD relevance	Evidence supporting neuroimmune modulation in depression	Experimental evidence	[149,150,151,152,153]
Adapted Cognitive Behavioral Therapy (CBT)	Cognitive restructuring adapted for ASD communication style	Increasing evidence in autistic adolescents and adults	Strong evidence in general MDD	Moderate to strong evidence when adapted	[7,154]
Social and Vocational Support Interventions	Reduce social isolation and structural stressors	Evidence suggests improved quality of life in autistic adults	Social support protective against depression	Moderate psychosocial evidence	[155]

**Table 4:** Mechanistic Subtypes and Targeted Intervention Hypotheses in ASD–Depression Comorbidity.

Mechanistic Subtype	Core Pathophysiology	Clinical Indicators	Targeted Intervention Hypothesis	References
Serotonergic Dysregulation Subtype	Altered SERT function, peripheral hyperserotonemia, monoaminergic imbalance	Mood lability, anxiety, sleep disturbance, irritability	Carefully titrated SSRIs; multimodal serotonergic modulation	[22,25,26,27,28,29,34,35,87]
Dopaminergic Reward Deficit Subtype	Mesolimbic reward circuit dysfunction; reduced dopaminergic signaling	Anhedonia, reduced motivation, restricted reward processing	Dopaminergic agents (e.g., bupropion); behavioral activation	[30,31,32,33,101]
Neuroinflammatory Subtype	Elevated IL-6, TNF-α; microglial activation; immune–brain axis dysregulation	Fatigue, cognitive slowing, treatment resistance	Anti-inflammatory approaches; curcumin; polyphenols; probiotics; postbiotics	[53,54,55,56,57,58,59,60,61,62,63,123,124,125,126,127,128,129,130,131,132,133,134,135,136,137,138,139,140,141,142,143,144,145,146,147,148]
HPA Axis Dysregulation Subtype	Chronic cortisol elevation; stress-induced plasticity loss	Burnout, emotional exhaustion, sleep disruption	Stress adapted psychotherapy; microbiota targeted modulation	[48,49,50,51,52,75,76,77,142]
Glutamatergic Dysregulation Subtype	NMDA receptor imbalance; excitation–inhibition disruption	Treatment-resistant depression; sensory hypersensitivity	NMDA modulators (ketamine, memantine)	[108,109,110,111,112,113,114,115]
Network Dysconnectivity Subtype	DMN–CEN–SN imbalance; fronto-limbic dysregulation	Rumination, social withdrawal, cognitive rigidity	TMS; theta-burst stimulation; neuromodulation	[36,43,116,117,118,119,120,121,122]

**Table 5:** Comparative Effectiveness of Therapeutic Strategies vs. SSRIs in ASD.

Strategy	Effectiveness Comparison to SSRIs	Unique Advantage/Clinical Note	Primary Risk/Disadvantage	References
SSRIs	Baseline	Standard treatment for stabilizing mood and repetitive behaviors.	High risk of behavioral activation (impulsivity, hyperactivity) or severe mania.	[100,101,102]
SNRIs	Alternative for TRD	Preferred when SSRIs are ineffective or fatigue/anhedonia are prevalent.	Sensitivity to side effects (GI issues, dizziness); risk of Serotonin Syndrome.	[106,109,110,111]
Atypical Antidepressants	Symptom-Specific	Bupropion addresses ADHD symptoms; Mirtazapine helps aggression/sleep.	Bupropion carries a risk of seizures and psychosis in prone individuals.	[101,102,106,112,113]
NMDA Modulators	Rapid & Targeted	Ketamine provides rapid onset; Memantine reduces irritability/social withdrawal.	Potential for ketamine abuse; unknown long-term effects on neurodevelopment.	[108,109,110,115]
TMS	Comparable Efficacy	Non-invasive; 50–60% response rate in treatment-resistant depression.	Sensory discomfort from noise/sensations; requires customized coil placement.	[117,121]
Probiotics	Adjunctive/Behavioral	Improves social responsiveness and anxiety; treats GI comorbidities.	Less evidence for treating “core” MDD compared to pharmaceuticals.	[144,147,148]
St. John’s Wort	Comparable (Mild/Mod)	Efficacy similar to SSRIs but with superior tolerability and lower dropout rates.	Significant risk of herb–drug interactions (Cytochrome P450 induction).	[129,130,131]

## Data Availability

The data presented in this study are available on request from the corresponding author.

## Abbreviations

The following abbreviations are used in this manuscript:

ASD	Autism Spectrum Disorder
5-HT	5 Hydroxytryptamine (Serotonin)
ACC	Anterior Cingulate Cortex
BDNF	Brain Derived Neurotrophic Factor
CBT	Cognitive Behavioral Therapy
CEN	Central Executive Network
CNV	Copy Number Variation
DLPFC	Dorsolateral Prefrontal Cortex
DMN	Default Mode Network
GWAS	Genome Wide Association Study
HPA	Hypothalamic–Pituitary–Adrenal axis
IL-6	Interleukin 6
MDD	Major Depressive Disorder
mTOR	Mammalian Target of Rapamycin
NMDA	N-methyl-D-aspartate
SERT	Serotonin Transporter
SN	Salience Network
SNRI	Serotonin Norepinephrine Reuptake Inhibitor
SSRI	Selective Serotonin Reuptake Inhibitor
TBS	Theta-burst stimulation
TMS	Transcranial Magnetic Stimulation
TNF-α	Tumor Necrosis Factor alpha
TRD	Treatment-resistant depression
